# Supplementary material for: A highly accurate platform for clone-specific mutation discovery enables the study of active mutational processes
Source: eLife. 2020 Apr 7;9:e55207. doi: 10.7554/eLife.55207 (PMC7228773; doi:10.7554/eLife.55207)
Supplement: Figure 2—source data 2. — Amplicon sequencing of the target sites was performed on the MiSeq platform. 3 out of the 14 targets appeared to have a high noise levels in the blood sample (highlighted in orange) and were therefore deemed inconclusive. Of the remaining 11 mutations only one did not seem to have any evidence in the bulk DNA sample of the PT2R tumor (highlighted in blue). VAWF: Variant Allele Well Fraction. [file elife-55207-fig2-data2.docx]

| Position | Mutation | Total Well Coverage | VAWF (%) | % Reads supporting the variant | |
| --- | --- | --- | --- | --- | --- |
|  |  |  |  | Blood gDNA | PT2R gDNA |
| chr1:43050977 | C>T | 45 | 33.33% | 0.03 | 0.22 |
| chr1:84044398 | T>C | 14 | 78.57% | 0.02 | 0.85 |
| chr1:168274409 | C>G | 15 | 40.00% | 0.33 | 0.49 |
| chr2:33010682 | A>G | 36 | 58.33% | 0.00 | 0.77 |
| chr2:123789390 | C>T | 28 | 46.43% | 0.00 | 0.27 |
| chr3:121522102 | C>G | 46 | 43.47% | 0.19 | 0.30 |
| chr3:129524269 | G>A | 20 | 45.00% | 0.00 | 0.31 |
| chr4:76733361 | C>T | 11 | 27.27% | 0.03 | 0.03 |
| chr4:167932516 | T>C | 33 | 39.40% | 0.02 | 0.09 |
| chr6:73146508 | C>G | 33 | 45.45% | 0.12 | 0.12 |
| chr15:59093127 | G>A | 18 | 61.00% | 0.00 | 0.85 |
| chr17:66520311 | C>T | 18 | 38.89% | 0.06 | 0.46 |
| chr17:66521158 | C>T | 22 | 31.82% | 0.01 | 0.09 |
| chr17:66523728 | C>T | 33 | 39.39% | 0.02 | 0.35 |

**Figure 2-source data 2. Targeted sequencing of some of the clone-specific variants identified in run D1111.** Amplicon sequencing of the target sites was performed on the MiSeq platform. 3 out of the 14 targets appeared to have a high noise levels in the blood sample (highlighted in orange) and were therefore deemed inconclusive. Of the remaining 11 mutations only 1 did not seem to have any evidence in the bulk DNA sample of the PT2R tumor (highlighted in blue). VAWF: Variant Allele Well Fraction.
