# Supplementary material for: A highly accurate platform for clone-specific mutation discovery enables the study of active mutational processes
Source: eLife. 2020 Apr 7;9:e55207. doi: 10.7554/eLife.55207 (PMC7228773; doi:10.7554/eLife.55207)
Supplement: Figure 2—source data 3. [file elife-55207-fig2-data3.docx]

| Position | Mutation | Total Well Coverage | VAWF (%) | % Reads supporting the variant | |
| --- | --- | --- | --- | --- | --- |
|  |  |  |  | Ascites gDNA | Blood gDNA |
| chr1:198871416 | C>G | 11 | 54.55 | 0.00 | 0.00 |
| chr1:205392201 | G>A | 26 | 38.46 | 0.02 | 0.00 |
| chr1:212152502 | C>A | 28 | 21.43 | 0.04 | 0.03 |
| chr2:165954947 | C>A | 14 | 42.86 | 0.01 | 0.02 |
| chr3:55936181 | C>A | 14 | 78.57 | 0.02 | 0.05 |
| chr3:80372726 | C>A | 9 | 66.67 | 0.01 | 0.02 |
| chr3:148085551 | C>T | 10 | 40.00 | 0.00 | 0.01 |
| chr3:166385800 | G>A | 15 | 46.67 | 0.00 | 0.01 |
| chr4:21723721 | C>T | 8 | 75.00 | 0.02 | 0.00 |
| chr4:114452967 | A>C | 16 | 68.75 | 0.00 | 0.00 |
| chr4:187590704 | G>T | 9 | 88.89 | 0.02 | 0.00 |
| chr5:24745295 | C>A | 11 | 36.36 | 0.01 | 0.01 |
| chr6:29409126 | C>T | 21 | 33.33 | 0.01 | 0.02 |
| chr6:89320707 | C>A | 7 | 57.14 | 0.01 | 0.02 |
| chr6:120042047 | C>A | 12 | 33.33 | 0.00 | 0.00 |
| chr7:11409022 | C>T | 9 | 55.56 | 0.01 | 0.03 |
| chr7:16023731 | C>A | 21 | 71.43 | 0.07 | 0.02 |
| chr7:140434437 | G>T | 28 | 39.29 | 0.01 | 0.00 |
| chr8:28991687 | G>T | 31 | 25.81 | 0.02 | 0.03 |
| chr8:47633895 | G>T | 25 | 16.00 | 0.02 | 0.02 |
| chr8:56078651 | G>T | 24 | 20.83 | 0.01 | 0.00 |
| chr8:133086518 | A>T | 16 | 31.25 | 0.03 | 0.01 |
| chr8:134979562 | G>T | 11 | 36.36 | 0.04 | 0.07 |
| chr11:5653934 | C>A | 26 | 19.23 | 0.01 | 0.03 |
| chr11:119730031 | C>T | 43 | 13.95 | 0.01 | 0.00 |
| chr12:128816427 | G>T | 9 | 55.56 | 0.01 | 0.02 |
| chr13:80467921 | G>T | 8 | 62.50 | 0.03 | 0.01 |
| chr15:34394338 | G>A | 10 | 60.00 | 0.01 | 0.01 |
| chr15:81086223 | C>T | 28 | 78.57 | 0.02 | 0.01 |
| chr16:80389783 | G>T | 11 | 72.73 | 0.04 | 0.02 |
| chr17:10440734 | C>A | 15 | 26.67 | 0.03 | 0.01 |
| chr17:57290575 | T>C | 28 | 35.71 | 0.00 | 0.00 |
| chr18:24891922 | C>A | 21 | 76.19 | 0.06 | 0.01 |
| chr18:34756888 | G>T | 23 | 78.26 | 0.04 | 0.05 |
| chr18:52260039 | G>T | 20 | 75.00 | 0.02 | 0.01 |
| chr21:40855807 | T>A | 10 | 70.00 | 0.01 | 0.01 |
| chr22:25928538 | A>T | 15 | 73.33 | 0.00 | 0.02 |

**Figure 2-source data 3 Targeted sequencing of some of the artefactual variants identified in run DE111.**
